# Recommendations of the Canadian Consensus Group on the Management of Chronic Myeloid Leukemia

**DOI:** 10.3747/co.v13i6.124

**Published:** 2006-12

**Authors:** P. Laneuville, M.J. Barnett, R. Bélanger, S. Couban, D.L. Forrest, D.C. Roy, J.H. Lipton

**Affiliations:** * Division of Hematology, McGill University Health Centre–Royal Victoria Hospital and McGill University, Montreal, Quebec; † University of British Columbia, Vancouver General Hospital, and British Columbia Cancer Agency, Vancouver, British Columbia.; ‡ Department of Hematology, Hôpital Maisonneuve–Rosemont and Université de Montréal, Montreal, Quebec.; § Blood and Marrow Transplant Program, Queen Elizabeth ii Health Sciences Centre and Dalhousie University, Halifax, Nova Scotia.; || Leukemia/bmt Program of bc, British Columbia Cancer Research Centre, and Vancouver Hospital and Health Sciences Centre, Vancouver, British Columbia.; # Cellular Therapy Laboratory and Program, Hôpital Maisonneuve–Rosemont and Université de Montréal, Montreal, Quebec; ** Chronic Myelogenous Leukemia Group, Allogeneic Blood and Marrow Transplant Unit, University Health Network–Princess Margaret Hospital, and University of Toronto, Toronto, Ontario

**Keywords:** Chronic myeloid leukemia, clinical practice guideline, imatinib

## Abstract

Chronic myelogenous leukemia (cml) is a disease characterized by the expression of Bcr/Abl, an oncogenic protein tyrosine kinase, and by evolution over time from a relatively benign chronic phase to a rapidly fatal cml blast crisis. Until recently, the standard of care included potentially curative therapy with allogeneic stem cell transplantation, available only to a minority (about 10%) of patients, or medical therapy with interferon-α with or without cytarabine, which helped to prolong the chronic phase of the disease in a minority of patients. The availability of imatinib mesylate, a selective inhibitor of Bcr/Abl approved by Health Canada in 2001, has profoundly altered the clinical and laboratory management of cml. This change in practice has been reviewed by the Canadian Consensus Group on the Management of Chronic Myelogenous Leukemia and has resulted in a new set of recommendations for the optimal care of cml patients.

## 1. INTRODUCTION

In 1924, the median survival time of patients with untreated chronic myeloid leukemia (cml) was about 2.4 years. Until the 1980s, cml was regarded as incurable and inexorably fatal. Within the past two decades, three pivotal discoveries have prolonged patient survival and led to cure in some patients 1:

- Allogeneic stem cell transplantation (allogeneic sct) holds the promise of cure for selected patients with cml, but a lack of suitable donors and the higher incidence of graft-versus-host disease in older individuals have thwarted efforts to extend this procedure to all patients 1.
- Interferon-α (ifnα) has expanded the therapeutic options for transplantation-ineligible patients. In a substantial minority, this agent clearly prolongs survival. However, this progress comes at a price. For more than 50% of patients, ifnα-related side effects are intolerable and lead to dose reduction 2,3.
- The introduction of imatinib mesylate has ushered in the new era of molecularly targeted therapy 1. A prototype for modern oncologic therapeutics, imatinib mesylate targets the pathogenic basis of cml by inhibiting the Bcr/Abl protein tyrosine kinase 4.

Patients with chronic-phase (cp) cml who fail standard treatment have a high response rate to imatinib 5. Such dramatic, convincing progress in treatment is rare 2. Once again, the therapeutic approach to cml is changing to keep pace with the rapid evolution of biotechnology.

### 1.1 Therapeutic Dilemma

The key question facing many Canadian hematologists and their patients is how to capitalize on the therapeutic progress of imatinib mesylate without forfeiting the life-prolonging effects of ifnα or the curative benefits of allogeneic sct.

No information on long-term survival with imatinib mesylate is available. However, observations of imatinib mesylate resistance in patients in CP cml and of residual disease in complete cytogenetic responders casts doubt on its ability to prolong survival in every cml patient 2. Ultimately, only time can answer outstanding questions about the median survival of patients on imatinib mesylate. In the interim, the lack of long-term clinical data contributes to uncertainty about how to manage cml.

To resolve this therapeutic dilemma, the Canadian Consensus Group on the Management of Chronic Myelogenous Leukemia (ccgm-cml), a group of Canadian specialists in cml, met to review the strength of evidence and to formulate a consensus on general recommendations for the management of cml. The ultimate goal was to develop an evidence-based guideline for use in Canadian clinical practice.

## 2. METHODOLOGY

In November 2003, more than 70 Canadian hematologists and oncologists attended a series of regional meetings with the intention of developing a consensus on recommendations for the standard treatment of cml. Three regional consultations were held in Montreal, Toronto, and Vancouver. At each meeting, Canadian specialists in the management of cml (Appendix A) participated in a series of interactive workshops and developed consensus positions on key management issues.

Before these meetings, key opinion leaders had been invited to review the medical literature and clinical data on specific topics for presentation to the group. These leaders reviewed entries to medline (1985 to November 2003), The Cochrane Library, cancerlit, and cmlsupport.com, plus relevant abstracts and reports from the proceedings of the 1998–2003 annual meetings of the American Society of Hematology (ash) and the American Society of Clinical Oncology (asco). In addition, the National Comprehensive Cancer Network, National Institute for Clinical Excellence, National Guidelines Clearing-house, Cancer Care Ontario, and Canadian Medical Association databases were searched for relevant information on clinical practice guidelines for cml.

Subsequently, a steering committee (Appendix B), composed of representatives from each regional meeting, met to share the recommendations of their respective meetings and to reach a national consensus on key issues related to the management of cml. These meetings were funded by an unrestricted educational grant from Novartis Canada Inc.

The steering committee graded the levels of evidence according to National Cancer Institute of Canada practices for the evaluation of scientific evidence (Appendix C). Whenever possible, the steering committee incorporated newly emerging clinical data (since November 2003) into the recommendations to ensure the most up-to-date approach to the management of cml.

This consultative process led to the recommendations that follow. The recommendations describe current best practice in the management of cml. This paper presents recommended procedures for diagnosis and patient monitoring, and optimal therapeutic strategies for pharmacotherapy and transplantation.

Given that the understanding of and clinical research into cml is rapidly evolving, particularly in terms of the long-term effects of imatinib mesylate and newer drugs, the steering committee wishes to emphasize that these recommendations must be revisited as new data emerge.

## 3. DISCUSSION

### 3.1 Epidemiology of CML

The annual incidence of cml in North America is 1–2 cases per 100,000 population, which represents about 15% of all cases of leukemia 6. The incidence is slightly greater in men than in women (1.35:1 ratio). Median age at diagnosis is 45–55 years 6. Between 12% and 30% of patients are 60 years of age or older 6. Based on this incidence and Canadian cancer statistics, about 550 new cases of cml are estimated to be diagnosed annually in Canada 7 **(Level III).**

Chronic myelogenous leukemia is an acquired disorder with no known inherited predispositions. Disease concordance is absent in monozygotic twins. Exposure to ionizing radiation, as documented in Japanese survivors of the World War ii nuclear bomb attacks, is the only well-established risk factor for the development of cml, but this cause is seldom implicated in newly diagnosed patients 1.

### 3.2 Causative Factors in CML

The disease is characterized by the presence of the Philadelphia (Ph) chromosome, a shortened chromosome 22. The Ph chromosome is created when an apparently spontaneous and reciprocal, but unequal, transfer of genetic material occurs between chromosomes 9 and 22, which break in the long arms at q34 and q11 respectively. This translocation fuses the *BCR* gene on chromosome 22 to sequences of the *ABL* proto-oncogene from chromosome 9. The *BCR/ABL* fusion gene expresses a 210-kDa Bcr/Abl protein tyrosine kinase 1.

Chronic myelogenous leukemia is believed to originate when a single hematopoietic stem cell acquires a Ph chromosome carrying the *BCR/ABL* fusion gene. This dominant oncogene confers a proliferative advantage on its progeny, which gradually displace normal hematopoietic stem cells 1,8. Expression of the Bcr/Abl protein tyrosine kinase is thought to be the initiating event in the genesis of cml. The oncoprotein supports the proliferation of malignant cell clones.

The cellular effects of *BCR/ABL* expression are pleiotropic and include mitogenic stimulation of growth-factor signal transduction pathways, inhibition of apoptosis 9, altered adhesion to and regulation by bone marrow stromal cells 10, functional activation of *ras* 11, impairment of the cellular response to genotoxic stress, and enhancement of the rate of secondary mutagenesis 12.

### 3.3 Definition, Classification, and Natural History

Various clinical definitions are used to categorize patients into three stages of cml: cp, accelerated phase (ap), and blast crisis (bc). Today, the most widely used definition is based on the percentage of blasts in bone marrow. This definition, employed by investigators in the iris trial [International Randomized Study of Interferon versus ST1571 (ST1571 being now known as imatinib mesylate)] 13, was adopted by consensus at the ccgm-cml meetings.

This definition simplifies the classification of cml set out in National Comprehensive Cancer Network (nccn) Clinical Practice Guidelines 14 It replaces outdated recommendations such as the International Bone Marrow Transplant Registry criteria, which include benchmarks referring to chemotherapy—an outdated treatment modality for most cml patients 14 It is not as strict as World Health Organization criteria 14, which define bc as ≥20% blasts in blood or marrow and which insist on cytogenetic evidence of clonal evolution. The latter requirement may delay treatment at Canadian sites that lack immediate access to cytogenetic evaluation.

About 85% of patients with cml present in cp, which is characterized by clonal granulocytic hyperplasia of relatively mature, functional cells 15. Signs and symptoms may include painful splenomegaly, fever, night sweats, anorexia and weight loss, anemia, hyperuricemia and gout, or complications arising from leucocytosis in individuals who have a very high peripheral blood cell count. All symptoms usually resolve with normalization of the peripheral blood count. In fact, up to half of patients today are asymptomatic and are diagnosed on routine blood testing, or when testing is done for other reasons 8,15.

Before the advent of ifnα and imatinib mesylate, orally administered busulfan and hydroxyurea were commonly used to induce hematologic remissions 15. Busulfan or hydroxyurea can normalize the number of peripheral blood granulocytes, but circulating cells almost always remain Ph chromosome–positive 8. More importantly, treatment with busulfan and hydroxyurea does not prevent the evolution of cml from a stable cp to ap and finally to bc 8,15.

The bc stage is invariably fatal, with a median survival in the range 3–6 months. The median duration of ap is in the range 6–9 months, but a substantial number of patients (25%–40%) may progress abruptly from cp to bc without a clearly defined intervening ap 8,15. About 5%–10% of patients typically progress from cp to bc in the first 2 years, with a rate of progression of 20% in subsequent years 16. Disease progression is accompanied by clonal evolution, characterized in most patients by the appearance of secondary cytogenetic abnormalities; altered gene methylation; and mutation, suppression, or overexpression of a number of genes 17,18.

#### Recommendation 1

The ccgm-cml recommends the following categorization of cml stages, as defined by the iris investigators 13 **(Level I.1iiDii)**:

- *Chronic phase* The presence of less than 15% blasts, less than 20% basophils, and less than 30% blasts plus promyelocytes in peripheral blood and marrow.
- *Accelerated phase* The presence of at least 15% blasts in blood or bone marrow, at least 30% blasts plus promyelocytes in blood or bone marrow, at least 20% peripheral basophils, or thrombocytopenia (platelets < 10×10^9^/L).
- *Blast crisis* The presence of at least 30% blasts in blood or bone marrow or extramedullary involvement—for example, chloromas.

### 3.4 Prognosis

A number of clinical prognostic scales have been developed to better estimate the prospective risk of cml progression and survival. Clinically, prognostic factors guide the selection of optimal treatment and the development of risk-adjusted treatments in patients awaiting therapy 19 **(Level II-3.3iiiA).**

The Sokal score was the first scale that achieved widespread use before the introduction of ifnα therapy. It works well as a prognostic discriminator of survival in patients treated with busulfan or hydroxyurea, but it is a poor discriminator in patients treated with ifnα.

The Sokal score categorizes patients into low [hazard ratio (hr): < 0.8], intermediate (hr: 0.8–1.2), or high risk (hr: >1.2) of death by rating these prognostic variables:

- Patient age
- Degree of splenomegaly
- Platelet count
- Percentage of peripheral blasts on a scale of increasing severity

Low-risk patients have a 2-year survival of 90%, a subsequent risk of less than 20% per year, and a median survival of 5 years. The high-risk group has a 2-year survival rate of 65%, a death rate of about 35% per year, and median survival of 2.5 years 3,20 **(Level II-3.3iiiA).**

The Sokal score is calculated for patients aged 5–84 years as:


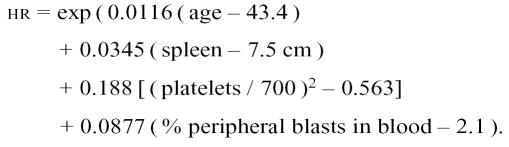


The Hasford prognostic score has proven to be more reliable for patients who choose ifnα 3,19 **(Level II-3.3iiiA)**. Until recently, Hasford was the most commonly used prognostic scoring system. It relies on these prognostic indicators:

- Patient age
- Blast count
- Basophil count
- Spleen size
- Eosinophil count
- Platelet count

It remains a reliable indicator of prognosis in the subset of patients on ifnα therapy. The Hasford score can be calculated for individual patients at the Web site www.pharmacoepi.de/cgi-bin/pharmacoepi/cmlscore.cgi.

The Sokal and Hasford prognostic scoring systems are both based on clinical and laboratory data obtained at the time of diagnosis; scores obtained after therapy initiation are less reliable. No prognostic scoring system has been specifically designed for patients on imatinib mesylate, although the Sokal and Hasford systems have both been used in major clinical trials of imatinib therapy 13. The Sokal system appears to have gained wider acceptance.

Marin *et al.* 21 devised a prognostic scoring system for patients on imatinib mesylate after failure of ifnα **(Level II-3.3iiiDi)**. In a sample of 145 patients, these authors showed that failure to achieve at least partial cytogenetic response and the presence of neutrophilia at 3 months were the only independent predictors of progression-free survival (pfs). They constructed a risk score of 0, 1, or 2 points that classifies patients into three prognostic groups. On this scale, patients score 1 point if they fail to achieve a partial cytogenetic response at 3 months and 1 point if they develop neutrophilia—defined as more than 1×10^9^ cells/L—between days 45 and 90 of therapy. The prognosis for survival at 2 years was 100% for the low-risk (0 points) group, 82% [95% confidence interval (ci): 70%–90%] for the intermediate-risk (1 point) group, and 40% (95% ci: 25%–58%) for the high-risk (2 points) group (*p* < 0.0001). The scoring system was validated in a small sample of 29 patients. Further validation of this prognostic scoring system is needed. At the time of writing, whether this scoring system can be applied reliably to patients on imatinib mesylate who have received no prior treatment with ifnα is unknown.

#### Recommendation 2

Until further investigation validates a more reliable prognostic score for patients who are considering imatinib mesylate, the ccgm-cml recommends the use of the Sokal index for newly diagnosed patients with cml.

### 3.5 Diagnosis and Baseline Investigations

At patient presentation, investigations should include assessment of prognosis with the Sokal index, a bone marrow (bm) biopsy, baseline bm G-banding cytogenetics, peripheral blood fluorescence *in situ* hybridization (fish), and quantitative real-time polymerase chain reaction (q-rt-pcr) determination. It is important to distinguish the latter test from *qualitative* or *semi-quantitative* pcr techniques.

The diagnosis of cml is established by any combination of positive results from bm cytogenetic studies, fish, and q-rt-pcr. Some of this testing appears redundant, but it serves different purposes.

Bone marrow morphology and blast counts remain the standard for assigning cml patients to the cp, ap, or bc stage of the disease.

The biopsy, besides characterizing cellularity, helps to determine if fibrosis is present at diagnosis.

Baseline bm cytogenetics are indicated to identify patients who may already show signs of clonal evolution at diagnosis. It also confirms whether the Ph chromosome is present or absent.

A baseline fish can determine if chromosome der9 deletions are present or absent. The presence of these deletions is a significant adverse prognostic factor in cp cml patients treated with ifnα 22, but appears to have less impact in patients treated with imatinib mesylate 23 **(Level II-2.3iiiA)**. Nevertheless, a baseline fish study will help to establish if fish can subsequently reliably be used to follow the cytogenetic response to treatment in the subset of patients who currently use or may cross over to ifnα therapy.

A q-rt-pcr should be performed at baseline. It will help to diagnose the rare cml patient who is Ph-negative by conventional cytogenetic analysis and also fish-negative. More importantly, it also provides baseline data for later comparative studies to monitor treatment response to imatinib mesylate. Most cp patients will achieve a complete cytogenetic response (ccr) on imatinib mesylate (79% at 31 months) 24 **(Level I-2.1iiDii)**. The q-rt-pcr is the only technique sufficiently sensitive to monitor and ensure that a biologic response to imatinib mesylate is sustained in ccr patients.

#### Recommendation 3

The ccgm-cml recommends the following investigations at diagnosis for all patients with suspected or confirmed cml:

- Assessment of prognosis (Sokal)
- Bone marrow aspirate and biopsy
- Baseline bone marrow cytogenetics
- Peripheral blood or bone marrow fish for deletions in chromosome 9
- Peripheral blood or bone marrow q-rt-pcr

### 3.6 Therapeutic Considerations for CML

Before developing their recommendations for treatment of cml, the ccgm-cml reviewed the following evidence-based data and considerations from clinical practice:

- Allogeneic sct for cml may be curative, but several difficulties independent of donor availability limit its application as universal therapy for cml. Many patients fail to qualify for transplantation because of age, health, disease status, and other factors. The risks of transplantation are relatively high and potentially life threatening.
- Medical therapy is able to prolong the duration of cp cml, reduce the rate of transformation, and improve the annual rate of survival. If tolerated by most patients, it may produce a greater impact on overall cml survival than does allogeneic sct, even if not curative.

#### 3.6.1 Allogeneic SCT

Allogeneic sct is the only known cure for cml. Until recently, it was the treatment of choice for patients 55 years of age and younger in generally good health who had a related donor matched for human lymphocyte antigens (hlas), or for those 40–45 years of age or younger with an hla-matched unrelated donor. These criteria, which determine eligibility for treatment, are used at most Canadian transplantation centres. Reduced-intensity allografting, which makes use of the graft-versus-leukemia effect of the allograft, is relatively new and may extend the age limit for transplantation to 79 years or more. Physicians should check with local transplant centres.

**FIGURE 1:**
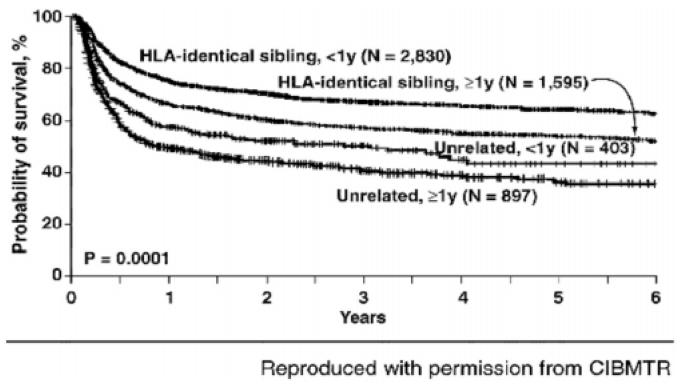
Probability of survival after 5725 allogeneic stem cell transplants for chronic-phase chronic myelogenous leukemia by donor type and disease duration, 1991–1997^3^. hla = human lymphocyte antigen

Data from the International Blood and Marrow Transplant Registry show that the 5-year survival for an hla-matched, related-donor allogeneic sct performed in cp cml less than 1 year after diagnosis ranges from 45% to 80% (see Figure 1) 3.

Survival is significantly lower if allogeneic sct is delayed beyond 1 year or if an hla-matched unrelated donor is used. Similarly, survival is considerably lower if cml progresses to ap before transplantation. Patients in bc seldom derive any benefit.

The choice of allogeneic sct in cp cml involves a tradeoff between exposure to early transplantation-related mortality and long-term disease-free survival. Moreover, the decision needs to be made early, when the patient is well, to minimize the chance that progression will occur and compromise the effectiveness of the procedure.

The risk:benefit ratio varies for individuals, depending on their specific disease-related and allogeneic sct–related risks. The most widely used method to evaluate risk factors and transplantation outcome in cml patients is described by Gratwohl *et al.* 25 **(Level II-3.3iiiA)**, based on an analysis of 3142 patients transplanted between 1989 and 1997 by the European Group for Blood and Marrow Transplantation (Table I). Gratwohl and colleagues developed a clinical scoring system, ranging from 0 to 7, to estimate the 5-year transplantation-related mortality and overall survival for individual patients (Table II).

Despite its effectiveness, allogeneic sct has had a relatively modest impact on cml survival. Because of age restrictions, absence of a suitable donor, health status, and other considerations, only about 10% of cml patients are eligible for the procedure. Survival in the range 80%–85% at 10 years has been reported by some institutions in young patients who are optimally conditioned with busulfan and cyclophosphamide therapy (as opposed to total body irradiation and cyclophosphamide), when the dose of busulfan is adjusted according to its measured clearance in individual patients 26 **(Level II-2.3iiA)**. These results, which can be achieved in only a minority of cml patients, are roughly equivalent to overall survival rates predicted for patients on imatinib mesylate therapy, which is feasible in most cml patients.

As Goldman *et al.* 27 indicate, the advent of imatinib mesylate has greatly complicated the decision-making process for patients who are weighing the risks and benefits of transplantation versus medical therapy. Two divergent approaches to managing the newly diagnosed patient with chronic cml have emerged. One approach is to offer all such patients a 2- to 3-month trial of imatinib mesylate to determine whether they will respond to treatment. Responders would continue on imatinib mesylate indefinitely, while eligible non-responders would proceed to allogeneic sct.

The other approach advocates the identification of low-risk, eligible patients for whom allogeneic sct could be confidently recommended as primary treatment 27. This group would likely include patients under 45 years of age with an hla-identical sibling donor and patients under 35 years of age with an hla-matched unrelated donor. For those in the latter category, the cytomegalovirus status of patient and donor must be taken into account. Goldman *et al.* 27 also recommend the use of prognostic scores such as the Sokal index as a guide, suggesting that, for patients with a higher probability of survival with medical therapy (that is, those in the Sokol “good risk” category), the upper age limit for transplantation be lowered by 10 years. Conversely, for patients in the Sokol “poor risk” category, the upper age limit could be increased by 10 years.

The nccn guideline recommends the discussion of allogeneic sct as a first-line therapy with eligible candidates 14. It points out that the widespread application of this potentially curative therapeutic option is limited by donor availability and high toxicity in older patients, which limits the age of eligibility to less than 65 years at many centres.

The France Intergroupe des Leucémies Myéloïdes Chroniques (filmc) recommends first-line allogeneic sct for young patients (<20 years) at low risk of disease progression 28.

The Ontario-based guidelines point out that the safety and efficacy of allogeneic sct has not been assessed in randomized clinical trials for the initial treatment of cp patients 29 It also notes that prior imatinib therapy does not appear to compromise allogeneic sct, except by delaying the procedure.

##### Recommendation 4

The option of allogeneic sct should be discussed with all transplantation-eligible patients. All transplantation-eligible patients should be referred to a transplantation centre for hla typing and matching of potential donors. Referral permits advanced preparation for allogeneic sct in the event that this option is chosen as first-line therapy or that imatinib mesylate therapy fails; in either case, the patient can proceed to transplantation in a timely manner. Allogeneic sct as first-line therapy is an acceptable option that may be selected by some patients based on personal preference after discussion of the pros and cons versus imatinib mesylate therapy. Patient choice is the determining factor in treatment decision-making.

#### 3.6.2 Interferon-α

Interferon-α (with or without cytarabine therapy) was the first agent to alter the natural history of cml and prolong survival, as compared with conventional therapy with busulfan or hydroxyurea 3. Until recently, most patients ineligible for allogeneic sct were offered ifnα therapy 3.

An important surrogate endpoint in clinical trials of ifnα was the degree of cytogenetic response—that is, reduction in the percentage of Ph-positive cells—observed in patients who were induced into hematologic remission. A major improvement in survival, up to 72% at 10 years, was observed in 5%–33% of patients, who achieved a ccr (0% Ph-positive cells) within 12–24 months 3. Patients with low-risk Sokal scores and a ccr within 12–24 months fared the best, with a 10-year probability of survival of 89% 3. Based on this evidence, ifnα became the first-line medical therapy for cml, despite a considerable increase in toxicity as compared with conventional therapy.

Interferon-α therapy has several drawbacks. Treatment for one or more years (12–24 months) is required to establish the presence and degree of cytogenetic response, owing to the slow kinetics of the biologic response to the drug 3, and therapy offers prolonged survival to a limited number of patients 3. Overall, cp patients with a low-risk Sokal score have a 10-year survival rate of about 40% 3. Tolerability is poor, and dose adjustment is required in more than 50% of patients 3. Up to 25% of patients discontinue treatment because of severe adverse effects 3.

The use of ifnα has not significantly altered the practice of offering allogeneic sct up front to eligible patients who have suitable donors. The modest survival benefit conferred by ifnα (except in the small percentage of patients who achieve ccr), together with the significant treatment-related toxicity, slow cytogenetic response, and controversial reports of prior ifnα treatment adversely affecting survival after allogeneic sct, have maintained the status quo for allogeneic sct–eligible patients.

The nccn no longer recommends the use of ifnα as initial therapy for cml; however, and although the evidence is limited, ifnα may be considered for second-line therapy in transplantation-ineligible patients who fail to respond to imatinib mesylate 14.

The Canadian representatives recommend that the cytogenetic response of patients who are initially treated with and remain on ifnα should be assessed. All patients who have less than a ccr should switch to imatinib mesylate. Patients who achieve a ccr on ifnα and who are tolerant of therapy should remain on ifnα and continue to be monitored every 3 months, as patients on imatinib mesylate are.

This recommendation differs slightly from the Ontario clinical practice guidelines, which suggest that it is reasonable for physicians to recommend a switch from ifnα to imatinib mesylate, because many patients are unlikely to remain on long-term ifnα, and the survival benefit with imatinib mesylate is not inferior to that of ifnα 29.

The National Institute for Clinical Excellence recommends that the decision to switch to imatinib mesylate should be based on drug tolerance, disease response, and patient preference 16. The filmc recommends the continuation of ifnα for stable patients with tolerable side effects 28.

##### Recommendation 5

The ccgm-cml recommends that patients on a pre-existing regimen of ifnα with or without cytarabine who experience intolerable side effects switch to imatinib mesylate. The ccgm-cml recommends the assessment of cytogenetic response in patients who are initially treated with and remain on ifnα. All patients with less than a ccr should be switched to imatinib mesylate. Patients who achieve a ccr on ifnα and who are tolerant of therapy should remain on ifnα and be monitored.

#### 3.6.3 Imatinib Mesylate

Clinical trials provide convincing evidence that imatinib mesylate, formerly called ST1571, an orally administered selective inhibitor of the Abl, Arg, Pdgfr, and Kit protein tyrosine kinases, is a useful treatment for all phases of cml 5,13,24 **(Level I.1iDii)**. Most significantly, it is superior to ifnα plus cytarabine in slowing disease progression and improving survival in cp patients 13 **(Level I.1iDii)**. Unfortunately, the results with more advanced disease are usually short-lived, and these patients should be considered for transplant at an earlier stage.

The pivotal clinical data come from the iris study, an open-label, phase iii, randomized study of 1106 patients with newly diagnosed cp cml 13 **(Level I.1iiDii)**. These *de novo* patients were randomized to receive imatinib mesylate (400 mg daily) or ifnα plus cytarabine arabinoside. After a median follow-up of 19 months, the estimated rate of minor cytogenetic response (mcr) at 18 months was 85.2% for imatinib mesylate and 22.1% for combination therapy (*p* < 0.001). The estimated rates of ccr at 18 months were 76.2% (95% ci: 72.5%–79.9%) for imatinib mesylate and 14.5% (95% ci: 10.5%–18.5%) for combination therapy (p < 0.001). Freedom from progression to ap or bc was estimated at 96.7% for imatinib mesylate and 91.5% for combination therapy (*p* < 0.001) 13 **(Level I.1iiDii)**.

An update of iris was presented at the asco Annual Meeting in June 2006 30,31. The trial is no longer randomized, because only 3% of the originally assigned cohort remain in the control arm; the remainder have crossed over to the imatinib arm. Hence, iris is now a long-term follow-up study. Reasons for crossover included intolerance, lack of response, and the fact that imatinib went on the market in the United States 13. No data for the control arm or crossover patients were presented at this update.

**FIGURE 2:**
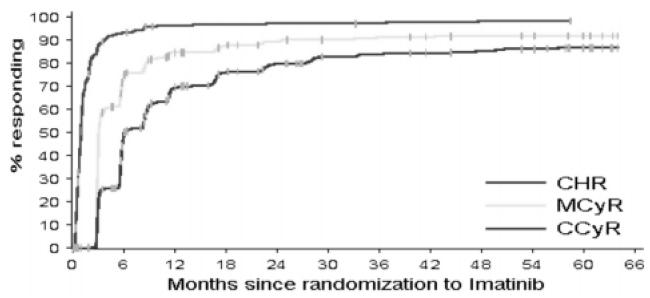
Progression-free survival rates and survival without accelerated phase or blast crisis with first-line imatinib [from the International Randomized Study of Interferon versus ST1571 (imatinib)] 31. CHR = complete hematologic response; MCyR = major cytogenetic response; CCyR = complete cytogenetic response.

**FIGURE 3:**
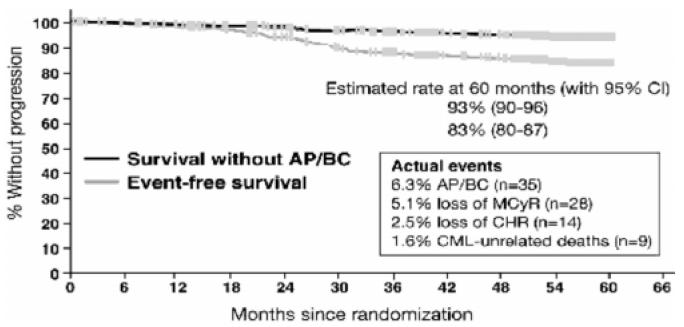
Estimated response to first-line imatinib. AP = accelerated phase; BC = blast crisis; CI = confidence interval; MCyR = major cytogenetic response; CHR = complete hematologic response; CML = chronic myelogenous leukemia. (Reproduced with permission from B.J. Druker.)

In the imatinib arm, at 60 months, 98% of patients had achieved a complete hematologic response (chr), 92% had an mcr, and 87% had a ccr (Figure 2) 30,31. At 60 months, 97% of patients who achieved a 3-log reduction in *BCR/ABL* transcripts at 12 months (ccr) remained progression-free. If they had achieved a <3-log but >2-log reduction in transcripts at 12 months (partial cytogenetic response), pfs was 93% 30,31. Among patients with no cytogenetic response at 12 months, pfs was 81% 30,31. Event-free survival was estimated at 83% (95% ci: 80%–87%) at 60 months, and pfs was estimated at 93% (95% ci: 90%–96%) at 60 months for patients on first-line imatinib (Figure 3) 30,31 **(Level II-2.3iDii)**.

Using a Kaplan–Meier model based on the intent-to-treat principle, the overall rate of survival for patients on initial imatinib therapy at 5 years was estimated at 89% (95% ci: 86%–92%), regardless of whether crossover had occurred 30,31. This analysis included deaths occurring after the discontinuation of imatinib therapy. Among patients initially randomized to imatinib, 57 deaths occurred: 14 after bm transplantation, 20 unrelated to cml, and 23 related to cml 30,31. When the analysis was censored for cml-related deaths, the estimated overall survival at 60 months was 95% 30,31 **(Level II-2.3iDii)**. International experts have agreed that this survival outcome is better than that of any other reported treatment for cp cml 32.

A major issue in treating newly-diagnosed cp patients with imatinib mesylate is how long to wait before determining an adequate response. The analysis of the iris cytogenetic data has provided evidence that the degree of cytogenetic response in imatinib mesylate–treated patients measured at 3, 6, and 12 months is predictive of pfs at 24 months, as had been surmised when the trial was first conceptualized 33 **(Level II-2.3iiiDii)**. The cytogenetic response observed at 3, 6, and 12 months can also predict the probability of achieving an mcr or ccr at 6, 12, and 24 months (Table III) 33 **(Level II-2.3iiiDii)**. Recent iris data reported at asco 2006 indicate that a late response to imatinib may occur and that the timing of the response does not affect its durability 30,31.

Recent evidence also suggests that achieving a ccr is an important factor that influences pfs. Among 370 patients who had achieved a ccr by q-rt-pcr at 3 months, those who had a major molecular response (mmr), defined as 3-log or greater reduction in *BCR/ABL* mrna levels, had a 100% probability of remaining progression-free at 24 months, regardless of medication 34 **(Level II-2.3iiiDii)**. Imatinib mesylate was more efficient than ifnα plus cytarabine was in eliciting a mmr 34 **(Level II-2.3iiiDii)**. At 12 months, about 39% of imatinib mesylate patients as compared with 2% of combination-therapy patients achieved a mmr 34 **(Level II-2.3iiiDii)**.

The 60-month iris data, presented at asco in June 2006, indicate that a greater degree of cytogenetic response appears to convey a greater degree of protection from progression to ap or bc, regardless of when the cytogenetic response is achieved 30,31. Patients who had a ccr early or at 12, 18, or 24 months achieved similar levels of protection against progression.

The effects of imatinib appeared to be durable: Patients who achieved a ccr within the first 24 months of imatinib therapy had a 99% survival rate at 60 months. No patient with a ccr and mmr at 18 months had progressed to ap or bc at 60 months 30,31. However, all patients with a ccr at 18 months did exceptionally well regardless of their molecular response; in patients with a <3-log reduction in *BCR/ABL,* the estimated rate of survival at 60 months was 98% 30,31. Notably, those who failed to achieve a ccr by 18 months had a significantly poorer outcome, with an estimated rate of survival of 87% at 60 months 30,31 **(Level II-2.3iiiDii)**.

Regarding how long to wait for an adequate response to imatinib, 64% of patients who achieved a partial cytogenetic response by 12 months achieved a ccr at 24 months. Half of patients with a partial response at 18 months later achieved a ccr 30,31. More than one third (36%) of patients with no cytogenetic response at 12 months eventually developed a ccr. Among those with no cytogenetic response at 18 months, 27% later developed a ccr 30,31 **(Level II-2.3iDiii)**.

Overall rates of disease progression were low, and the rate of any progression for all patients on initial imatinib therapy declined over time (Table IV) 30,31 **(Level II-2.3iDii)**. This rate of transformation is a major improvement over the traditional rate of 10%–15% observed in patients treated with conventional therapy and the 5%–10% observed in patients treated with ifnα plus cytarabine.

The overall risk of transformation to ap or bc for patients on first-line imatinib was associated with the patient’s level of response, regardless of the timing of response achievement. Of particular interest, in patients who achieved a ccr on imatinib, the risk of any progression declined over time to less than 0.5% at 5 years 30,31. For patients on first-line imatinib, the annual percentages of progression to ap or bc declined from 2.1% in the first year to 0.8% in the second year, 0.3% in the third year, and 0% in the fourth year after achieving a ccr 30,31. The timing of the ccr appeared to have no impact on this result. Once ccr was achieved, the longer the patient remained on imatinib, the lower became the risk of any progression over time **(Level II-2.3iDii)**.

Evidence from iris clearly shows that imatinib mesylate is more effective than ifnα plus cytarabine as first-line therapy for newly diagnosed patients with cp cml 13,31,32 **(Level I.1iDii)**. Imatinib mesylate prolongs pfs and elicits a beneficial cytogenetic response in more patients without the toxicity of ifnα 13,31,32 **(Level I.1iDii; Level II-2.3iiiDii)**. Based on this evidence, the ccgm-cml reached a consensus regarding the use of imatinib mesylate as first-line therapy in this patient group. Data from q-rt-pcr showing that the extent of molecular response is predictive of pfs and can be evaluated as early as 3 months further supports the group’s position^27^ **(Level I.1iDii)**.

The nccn, the National Institute for Clinical Excellence, the filmc, Ontario-based guidelines, and the European LeukemiaNet expert panel recommend the use of imatinib mesylate as first-line therapy in newly diagnosed patients with cp, Ph chromosome–positive cml 14,16,28,29,32. The European LeukemiaNet recommends a trial of imatinib mesylate for any patient with newly diagnosed cp cml regardless of risk, given that a patient’s early response to imatinib can either reinforce or weaken the indication for allogeneic sct 32. Imatinib is also recommended as second-line therapy for patients who are refractory or intolerant to ifnα and for previously untreated patients with disease progression 14,29.

The best outcome for allogeneic sct is a 5-year survival rate of 75%–80% 14,35. Unfortunately, this result can be achieved only in a minority of cml patients. It is roughly equivalent to the overall survival rate predicted for patients on imatinib mesylate therapy, which is feasible in most cml patients.

The greater efficacy of medical therapy with imatinib mesylate has clearly shifted the established risk:benefit ratio with respect to allogeneic sct. Much discussion has ensued internationally and within the ccgm-cml consultative meetings about how to alter pre-existing recommendations for cml patients. At the regional and steering committee meetings, two major approaches, both of which accepted the premise that the survival benefit conferred by imatinib mesylate will likely be sustained on long-term follow-up, were considered.

The first approach is to redefine the indications for allogeneic sct to select younger patients who are at lowest risk for complications. The second is to offer imatinib mesylate as first-line therapy to all patients, to monitor the response to treatment, and to reserve allogeneic sct for patients who fail to respond to a degree predictive of prolonged disease-free survival. Patient preference between these two approaches, after full discussion of the relative risks and benefits, is to be respected and will often dictate the choice of treatment.

This choice was supported by evidence that the cytogenetic response to imatinib mesylate can be determined as early as 3 months after the start of treatment, well within the 1-year limit from diagnosis for optimal transplantation. Another key factor that contributed to this position was the comparison between the numbers of patients who are eligible and who stand to benefit from either treatment.

Many Canadian representatives strongly believe that it is essential to discuss the option of transplantation with their patients and to refer, at the time of diagnosis, any transplantation-eligible patient to a transplantation centre for standard evaluation. For any patient who fails to respond to imatinib mesylate, pre-assessment of transplantation risk will guide the decision to pursue transplantation or an alternative medical therapy, reducing further delays to transplantation. Having sibling matches or unrelated donors identified beforehand will reduce the delay significantly.

##### Recommendation 6

For all newly diagnosed patients with chronic-phase cml who do not elect related-donor allogeneic sct as first-line therapy, imatinib mesylate is recommended.

#### 3.6.4 Initiation of Imatinib

Imatinib mesylate was approved by Health Canada in late 2001 for newly diagnosed, cp cml patients at a dose of 400–600 mg daily and is now approved for patients in ap and bc at a dose of 600–800 mg daily. Health Canada approved a dose increase to 600 mg or 800 mg from 400 mg daily in adult cml patients with cp disease, and practitioners may consider 600 mg to 800 mg (given as 400 mg twice daily) in adult cml patients in ap or bc in the absence of severe adverse drug reactions and severe non-leukemia-related neutropenia or thrombocytopenia in the following circumstances:

- Disease progression (at any time)
- Failure to achieve a satisfactory hematologic response after at least 3 months of treatment
- Failure to achieve a cytogenetic response after 12 months of treatment
- Loss of a previously achieved hematologic or cytogenetic response 36

Imatinib mesylate (400 mg daily) is effective in cp patients after failure of ifnα therapy, but few patients achieve a molecular remission. At least one study of 36 patients has shown that high-dose imatinib mesylate (up to 800 mg daily) induces a ccr in most patients in cp after ifnα failure. The ccr was accompanied by a high rate of molecular remission 37 **(Level II-2.3iiiDiii)**.

In a study of 108 newly diagnosed patients in cp, Cortes *et al.* 38 **(Level II-2.3iiiDiii)** reported at ash 2003 that, as compared with the 400 mg daily dose, the 800 mg daily dose results in higher rates of ccr and molecular remission, with some increase in myelosuppression.

Higher doses of imatinib mesylate may overcome disease-poor response to conventional doses (400 mg daily) in cp patients. Limited experience suggests that up to one third of cp patients will achieve a major cytogenetic response with dose escalation 5. Ontario-based guidelines recommend dose escalation to 800 mg daily in cp patients who do not achieve a chr after 3 months—or at least an mcr after 12 months of therapy 29. The nccn recommends dose escalation to 600–800 mg daily after 6 months, as tolerated, in patients with a ccr, a partial cytogenetic response, or an mcr, and 800 mg daily as one therapeutic option in patients with no cytogenetic response 14.

In one study of 34 patients treated for cytogenetic resistance or relapse, 56% achieved a complete or partial cytogenetic response to higher doses (600–800 mg daily) of imatinib mesylate 39 **(Level II-2.3iiiDiii)**. Among 20 patients who had hematologic resistance or relapse, 65% achieved a complete or partial hematologic response 39 **(Level II-2.3iiiDiii)**. Doses of 800 mg daily are less well tolerated, with a higher incidence of fluid retention, skin rashes, and muscle cramps 5.

Preliminary research by Kantarjian *et al.* 40 **(Level II-1.2A)** indicates that a higher starting dose of 800 mg daily of imatinib mesylate elicits a significantly higher ccr, mmr, and complete molecular response in patients with Ph-positive cp cml than does a starting dose of 400 mg daily. Adverse events were similar in type and frequency for both dosages; however, at least one third of patients taking 800 mg daily required a dose reduction within 3–12 months.

At ash 2004, Cortes *et al.* 41 reported that initiating high-dose [hd (800 mg daily)] imatinib therapy results in higher rates of ccr and molecular remission in treatment-naïve cp patients than does standard-dose [sd (400 mg daily)] therapy **(Level II- 2.3iDii)**. In that study, 222 patients were allocated among three clinical trials: one trial of sd therapy (*n* = 50) and two with a hd regimen (*n* = 172). The estimated pfs at 12 months was 92% in the sd trial and 99% in the hd trials (*p* = 0.42). Patients on hd therapy had some increase in myelosuppression, as shown by these findings: grade 3 or worse anemia (7% on hd vs. 4% on sd), neutropenia (39% vs. 20%), and thrombocytopenia (27% vs. 12%). At 12 months, more patients in hd trials required dose reduction (36% vs. 14%).

In ap and bc studies to date, the main impact of higher imatinib doses is on time-to-progression and early survival 5.

After reviewing the evidence, the ccgm-cml reached a consensus that the minimum starting doses of imatinib mesylate should be 400 mg daily in patients in cp, 600 mg daily in patients in ap, and up to 800 mg daily in patients in bc.

##### Recommendation 7

The ccgm-cml recommends the following *minimum* starting dosages of imatinib mesylate for patients with cml:

- *Chronic phase* 400 mg daily
- *Accelerated phase* 600 mg daily
- *Blast crisis* Up to 800 mg daily

In patients who switch to imatinib mesylate after failure of ifnα with or without cytarabine, the ccgm-cml recommends a starting dose of 400 mg daily of imatinib mesylate.

#### 3.6.5 Monitoring Patient Response to Imatinib Mesylate

The ccgm-cml representatives felt that it was important to develop specific guidelines for patient monitoring to track therapeutic efficacy and development of resistance in patients on imatinib mesylate. The representatives strongly felt that a lack of routine monitoring compromises patient care, because decisions about adjusting or choosing alternative therapy cannot be made in a timely fashion to benefit patients.

Disease response to imatinib mesylate should be evaluated at 3-month intervals by q-rt-pcr or fish. Annual bm cytogenetic analysis should be performed in patients to detect evidence of clonal evolution and to document the presence of secondary cytogenetic changes that may be present in patients who achieve ccr. The appearance of secondary chromosomal abnormalities is often transient, and their prognostic significance has yet to be determined 15.

The Canadian representatives defined the major treatment-response milestones for patients in cp as the achievement of chr at 3 months, mcr at 12 months, ccr at 18 months, and mmr at 24 months. Patients who reach all of these milestones should continue on the same dose of imatinib mesylate as long as their response is sustained. At present, no step-down protocols have been developed for long-term maintenance therapy with imatinib mesylate. Failure to achieve the recommended therapeutic milestones or loss of responsiveness to imatinib mesylate on continued monitoring indicates a need to consider a change of therapy.

While acknowledging that the optimal guidelines for imatinib mesylate monitoring are unclear, the nccn suggests that cytogenetic testing may begin as early as 3 months after the start of therapy 14. In Ph-positive patients, bone marrow cytogenetics are recommended at 6 and 12 months after the start of therapy. After the patient achieves a ccr, the nccn recommends fish or q-rt-pcr to monitor patients every 3–6 months. If the patient has a >1-log increase on q-rt-pcr or a positive fish, bone-marrow cytogenetics are recommended. If no sign of disease progression is found, annual bone-marrow cytogenetics are recommended to detect clonal abnormalities 14.

The expert panel of the European LeukemiaNet recommends evaluation of hematologic response every 2 weeks until a chr is achieved and confirmed. They recommend cytogenetic evaluation before treatment, at least every 6 months until a ccr is achieved and confirmed, and then once every 12 months. They recommend checking the patient’s molecular response every 3 months 32. After the patient attains a mmr, conventional cytogenetic evaluations may be performed less frequently, depending on the patient’s clinical, hematologic, and molecular findings 32. The expert panel recommends fish only prior to treatment to identify Ph-negative patients 32. They state that chr, ccr, and mmr must be confirmed on two subsequent occasions 32.

##### Recommendation 8

The ccgm-cml recommends the following tests and frequency of testing to monitor hematologic, cytogenetic, and molecular response in all patients on imatinib mesylate and to guide therapeutic decision-making:

- bm cytogenetic testing
- At diagnosis
- Annually, if a major cytogenetic response is maintained
- q-rt-pcr
- At diagnosis and every 3 months (fish may be substituted every 3 months until ccr); q-rt-pcr and fish should be performed at standardized laboratories
- If a ≥ 0.5-log increase occurs, the test should be repeated within 4 weeks; mutational analysis is recommended
- *ABL*-kinase sequencing for mutations
- At confirmed increase of 0.5 log in the q-rt-pcr; *ABL* sequencing should be performed at standardized laboratories

The ccgm-cml recommends the achievement of the following therapeutic milestones, to indicate the successful progression of imatinib mesylate therapy in cp patients with cml:

- chr at 3 months
- Major cytogenetic response at 12 months
- ccr at 18 months
- mmr at 24 months

Failure to achieve these therapeutic milestones within the specified time limits indicates a need to reconsider the therapeutic strategy. The ccgm-cml recommends these alternatives:

- Allogeneic sct, if an option
- Increasing the dose of imatinib mesylate up to 800 mg daily

On continued lack of response despite maximum dose escalation, discontinuation of imatinib mesylate treatment and consideration of alternative therapy is justifiable. Once the milestones have been achieved, treatment should be maintained indefinitely as long as the patient continues to respond.

#### 3.6.6 Emergence of Resistance in Imatinib Mesylate-Treated Patients

Resistance to imatinib mesylate has emerged in a proportion of patients with cml who are treated with this drug as first-line therapy. Acquired resistance to imatinib mesylate is almost always associated with reactivation of Bcr/Abl kinase activity 42. In most cases, resistance is attributable to the presence of point mutations in the *BCR/ABL* kinase domain 43. Most patients who are prospectively monitored by q-rt-pcr and who have as little as a doubling in the level of *BCR/ABL* transcripts are shown to harbour *ABL*-kinase mutations 44 **(Level II-2.3iiiDiii)**.

A spectrum of *ABL*-kinase mutations has been described that variably abrogates the binding of imatinib mesylate and confers partial-to-complete resistance to imatinib mesylate. The presence of *BCR/ABL* kinase domain mutations has important prognostic and therapeutic implications. The location of mutations—within rather than outside the P-loop of the kinase domain—appears to have a major impact on the natural history of the disease 45 **(Level II-2.3iD)**. Patients with mutations in the P-loop have a particularly poor prognosis 43,44.

In an Australian study, 12 of 13 patients (92%) with P-loop mutations died within a median of 4.5 months after mutation detection, but only 3 of 14 patients (21%) with non-P-loop mutations had died after a median follow-up of 11 months 44. In that study, a longer time from diagnosis to the start of imatinib therapy and a failure to achieve an mcr by 6 months were associated with a higher incidence of mutation. The development of *ABL* mutations in cml patients confers varying degrees of resistance to imatinib mesylate, adversely affecting survival 45.

For optimal clinical management of patients on imatinib mesylate, early detection of treatment-resistant *ABL* mutations is crucial. Testing for *ABL* mutation should be requested for all cp cml patients who fail imatinib mesylate therapy (see the next subsection). Mutation testing should be restricted to a limited number of specialized laboratories. Routine screening of other responding or stable patients should not be done, because these patients almost never have a mutation.

#### 3.6.7 Failure of Imatinib Mesylate Therapy

In the event that therapeutic milestones are not reached or that disease progression is documented by q-rt-pcr or fish, transplantation eligibility becomes a major variable in decision-making. Canadian representatives agreed that all patients who fail imatinib mesylate therapy and who are eligible for transplantation should be referred for allogeneic sct.

Ontario-based guidelines suggest that, for transplantation-eligible patients who may choose allogeneic sct as second-line therapy in the advent of imatinib mesylate failure, cytogenetic analysis should be performed within 12 months of the start of imatinib therapy 29.

The filmc recommends allogeneic sct for eligible patients who have no cytogenetic response to imatinib mesylate at 3 months or who have a hematologic relapse within 12 months (5% of patients). They feel that the absence of a ccr at 12 months justifies discussion of allogeneic sct in transplantation-eligible patients under the age of 50 years 28. Similarly, the nccn recommends bm transplantation, if feasible, when no hematologic response to imatinib mesylate has occurred at 3 months 14.

The expert panel of the European LeukemiaNet defines the failure of imatinib at various stages of treatment: no hematologic response at 3 months, no cytogenetic response or partial hematologic response at 6 months, less than partial cytogenetic response at 12 or 18 months, and loss of chr, ccr, or development of mutation at any time 32.

In transplantation-ineligible patients who do not have P-loop *ABL* mutations or who have mutations associated with only partial resistance, the recommended approach is to escalate the dose of imatinib mesylate to 800 mg daily in an attempt to re-establish responsiveness. This practice is supported by evidence of a greater, more rapid response in patients who are initially treated with doses of imatinib mesylate greater than 400 mg daily and, more importantly, after dose escalation, of salvage of up to 50% of patients who initially progress on 400 mg daily 38.

Failing this strategy, transplantation-ineligible patients could be treated with ifnα with or without cytarabine or on a phase ii protocol of newer generation drugs, other experimental drugs, immunologic manipulations, or combination therapy, if available. Patients who fail both medical treatments should be offered palliative therapy with hydroxyurea.

##### Recommendation 9

The ccgm-cml recommends the following definition of disease progression in *compliant* patients on imatinib mesylate therapy:

- Transformation from cp to ap or bc
- Cytogenetic (clonal) evolution in Ph-positive cells
- Loss of ccr
- Confirmed increase of 0.5 log or more (q-rt-pcr) for patients in ccr or better
- Detection of *ABL* mutations with loss of response

For patients in whom imatinib mesylate fails, the ccgm-cml recommends these treatment strategies:

- Allogeneic sct for all transplantation-eligible patients
- Dose escalation up to 800 mg daily of imatinib mesylate in transplantation-ineligible patients who do not have *ABL* mutations that confer complete resistance to imatinib mesylate
- Therapy with ifnα (with or without cytarabine) for transplantation-ineligible patients who fail to respond to dose escalation within 3 months or who have mutations of *ABL* that confer resistance
- Institutional research board–approved therapeutic protocols for clinical trials of new, experimental agents^a^
- Treatment with hydroxyurea or busulfan in patients in whom ifnα may be deemed inappropriate^a^

### 3.7 Outstanding Issues

The recommendations of the ccgm-cml should lead to a major improvement in the treatment of patients with cml. For the few patients who are eligible for allogeneic sct, the recommendation for first-line therapy strikes a better balance—avoiding premature mortality and morbidity, while still providing the transplantation option for those who are unlikely to benefit from medical therapy or who choose a more aggressive approach up front.

The key to successful implementation of the guidelines in Canada is the availability of adequate laboratory support to monitor patient response. Without rigorous monitoring, a substantial number of patients may, over time, receive expensive and ineffective therapy, and transplantation-eligible patients may miss the opportunity to receive potentially lifesaving treatment.

Consequently, one of the ccgm-cml’s strongest recommendations is that these guidelines be implemented concurrently with the deployment of readily available standardized fish and q-rt-pcr testing for all Canadian patients. A total of seven standardized laboratories now provide q-rt-pcr cml testing in Canada. Another program to standardize up to 13 q-rt-pcr laboratories across Canada is underway. Efforts are also currently being made to establish long-term funding for Canadian referral centres to provide these services for all patients as the availability of this technology becomes more widespread.

#### Recommendation 10

The ccgm-cml strongly recommends standardized q-rt-pcr testing for all Canadian patients with cml. Mutational analysis should be regionalized in even fewer centres.

## 4. DISCLOSURE

The following physicians disclosed that they have received honoraria from Novartis Pharmaceuticals Canada Inc. for consultancy: Pierre Laneuville, Jeffrey H. Lipton, Michael Barnett, Robert Belanger, Stephen Couban, Donna Forrest, Denis-Claude Roy. Dr. Lipton also disclosed that he has received honoraria from Novartis Pharmaceuticals Canada Inc. as a participant in a speaker’s bureau.

## Figures and Tables

**TABLE I:** Calculation of risk factor score for transplantation patients (Grawohl *et al.*) 25

	*Points*		
	*0*	*1*	*2*
Stage	CP1	AP	BP≥CP2
Patient age (years)	<20	20–40	>40
Interval (months)	<12	>12	—
Patient/donor	Other	M/F	—
Donor	SD	UD	—

CP1 = first chronic phase; AP = accelerated phase; BP = Blast phase; CP2 = second chronic phase; M/F = male/female; SD = sibling donor; UD = unrelated donor.

**TABLE II:** Calculation of prognosis at 5 years by risk factor score (Gratwohl *et al.*) 25

*Score*	*Transplantation-related mortality (%)*	*Overall survival (%)*
0–1	20	70
2	30	60
3	45	50
4	50	40
5–7	70	20

**TABLE III:** Probability of achieving a complete cytogenetic response (ccr) and a minor cytogenetic response (mcr) at 6, 12, and 24 months by cytogenetic response at 3, 6, and 12 months

	*Response level*			
	*>95%*	*66%–95%*	*36%–65%*	*1%–35%*
*Cytogenetic response at 3 months:*				
Probability (range) of achieving a ccr at				
6 Months	3 (0–10)	8 (0–20)	11 (0–22)	55 (47–63)
12 Months	25 (9–41)	29 (10–48)	37 (21–54)	84 (78–90)
24 Months	48 (30–66)	56 (33–79)	61 (44–78)	91 (86–96)
Probability (range) of achieving an mcr at				
6 Months	9 (0–20)	38 (18–57)	60 (43–77)	
12 Months	47 (29–65)	60 (39–80)	83 (70–96)	
24 Months	57 (39–75)	70 (50–90)	86 (74–98)	
*Cytogenetic response at 6 months:*				
Probability (range) of achieving a ccr at				
12 Months	7 (0–20)	19 (0–38)	5 (0–16)	59 (48–71)
24 Months	14 (0–32)	46 (20–72)	50 (26–73)	77 (67–87)
Probability (range) of achieving an mcr at				
12 Months	13 (0–29)	50 (25–75)	79 (60–98)	
24 Months	34 (9–60)	64 (40–89)	93 (80–100)	
*Cytogenetic response at 12 months:*				
Probability (range) of achieving a ccr at				
24 Months	9 (0–27)	14 (0–41)	20 (0–56)	57 (36–71)
Probability (range) of achieving an mcr at				
24 Months	27 (0–54)	14 (0–41)	67 (28–100)	

**TABLE IV:** Annual event rates in patients on initial imatinib therapy 30,31

*Year*	*All events (%)*^a^	*Progression toap*/*bc(%)*
1	3.3	1.5
2	7.5	2.8
3	4.8	1.6
4	1.5	0.9
5	0.9	0.8

a All deaths or loss of response, including progression to accelerated phase (ap) or blast crisis (bc).

## Footnotes

The efficacy of ifnα after imatinib mesylate failure is unknown; no data are available.

## APPENDIX A PARTICIPANTS IN REGIONAL CONFERENCES

**Thierry Alcindor** **md abim**

Specialty: Internal Medicine, Hematology, Oncology

Medical Oncologist, Montreal General Hospital and Royal Victoria Hospital

Assistant Professor, Departments of Medicine and Oncology, McGill University

Montreal, Quebec

**Joffre Allard** **md**

Specialty: Internal Medicine (QC), Hematology (QC), Medical Oncology

Active Staff Member, Department of Hematology, Centre Hospitalier Regional de Rimouski

Rimouski, Quebec

**Peter Anglin** **md frcpc**

Specialty: Internal Medicine, Hematology, Medical Oncology (Clinical Hematology)

Active Staff Member, The Credit Valley Hospital Consultant, University Health Network—Princess Margaret Hospital Site

Courtesy Staff, Dufferin–Caledon Healthcare Corp.

Instructor, University of Toronto

Toronto, Ontario

**Nizar Bahlis** **md**

Specialty: Internal Medicine, Hematology, Oncology

Assistant Professor, University of Calgary and Tom

Baker Cancer Centre

Calgary, Alberta

**Isabelle Bence–Bruckler** **md frcpc**

Specialty: Internal Medicine, Hematology (Bone Marrow Transplantation)

Staff Member, Hematology, The Ottawa Hospital—General Campus

Associate Professor, Department of Medicine, University of Ottawa

Ottawa, Ontario

**Normand Blais** **md frcpc**

Specialty: Internal Medicine, Hematology, Medical Oncology

Hematologist and Medical Oncologist, Department of Medicine, CHUM—Hôpital Notre-Dame

Laval, Quebec

**Lambert Busque** **md frcpc**

Specialty: Internal Medicine, Hematology

Staff Member, Hôpital Maisonneuve–Rosemont

Director, Molecular Hematology

Associate Professor, Department of Medicine, Université de Montréal

Montreal, Quebec

**Stephen Caplan** **md frcpc**

Specialty: Hematology (Lymphoma)

Director, Division of Hematology, Sir Mortimer

B. Davis Jewish General Hospital

Associate Professor, Departments of Medicine and Oncology, McGill University

Montreal, Quebec

**Hak Ming Chiu** **md frcpc**

Specialty: Hematology, Internal Medicine, Medical Oncology

Active Staff Member, Oshawa Lakeridge Health Corporation—Oshawa Site

Oshawa, Ontario

**Kathy Chun** **p****h****d fccmg**

Specialty: Cancer Cytogenetics

Assistant Professor, Department of Laboratory Medicine and Pathobiology, University of Toronto

Head, Cancer Cytogenetics, University Health

Network Toronto, Ontario

**Denis Cournoyer** **md frcpc**

Specialty: Hematology, Internal Medicine

Staff Member, cusm–Montreal General Hospital

Associate Professor, Departments of Medicine and Oncology, McGill University

Montreal, Quebec

**Nazim Damji** **md frcpc dabim**

Specialty: Hematology, Oncology, Internal Medicine

Staff Member, Trillium Health Centre—Mississauga Site

Etobicoke, Ontario

**Robert Delage** **md frcpc**

Specialty: Hematology, Internal Medicine

Active Staff Member, Centre Hospitalier Affilié

de Québec—Hôpital du St-Sacrement

Associate Professor, Université Laval

Sainte-Foy, Quebec

**Yvan Drolet** **md**

Specialty: Hematology (QC), Medical Oncology (QC), Pediatrics

Staff Member, Centre Hospitalier Universitaire de

Québec—Hôtel-Dieu de Québec

Clinical Professor, Université Laval

Quebec City, Quebec

**Peter Duggan** **md frcpc**

Specialty: Hematology, Internal Medicine

Staff Member, Health Sciences Centre

St. John’s, Newfoundland

**Alain Filion** **md frcpc**

Specialty: Hematology, Internal Medicine, Medical Oncology (Hematology/Oncology)

Staff Member, Department of Hematology/Oncology, Centre Hospitalier Universitaire de Québec—Hôtel-Dieu de Québec

Hematology Oncologist, Hôtel-Dieu de Lévis

Quebec City, Quebec

**Kuljit Grewal** **md frcpc**

Specialty: Hematology, Internal Medicine

Chief Division of Hematology, Department of Medicine, Health Sciences Centre

Associate Professor, Department of Medicine, Memorial University

St. John’s, Newfoundland

**Marlene Hamilton** **md frcpc**

Specialty: Hematology, Internal Medicine, Medical Oncology

Assistant Professor, University of Alberta

Edmonton, Alberta

**Wanda Hasegawa** **md frcpc**

Specialty: Internal Medicine, Hematology (Bone Marrow Transplant)

Staff Physician, Royal University Hospital

Clinical Associate Professor, University of Saskatchewan

Saskatoon, Saskatchewan

**Josée Hébert** **md frcpc**

Specialty: Hematology, Internal Medicine

Staff Member, Hôpital Maisonneuve–Rosemont

Montreal, Quebec

**Donna Hogge** **md Phd frcpc**

Specialty: Hematology, Internal Medicine (Hematology/Oncology)

Active Staff Member, Vancouver Hospital and Health Sciences Centre, British Columbia Cancer Agency—Vancouver Cancer Centre

Clinical Professor, Department of Medicine, University of British Columbia

Vancouver, British Columbia

**Kang Howson–Jan** **md frcpc**

Specialty: Internal Medicine (Bone Marrow Transplant)

Staff Member, Department of Hematology, London Health Sciences Centre—Victoria Campus Site Assistant Professor, University of Western Ontario Consultant, London Regional Cancer Centre

London, Ontario

**Sabir Hussain** **md frcpc**

Specialty: Internal Medicine, Hematology, Oncology

Staff Member, Department of Oncology, Dr. Everett Chalmers Regional Hospital (SH)

Fredericton, New Brunswick

**Suzanne Kamel–Reid** **P****h****d**

Director, Molecular Diagnostics, Department of Pathology, The University Health Network

Head, Genetics Program, Toronto Medical Laboratories/UHN

Scientist, Ontario Cancer Institute

Professor, Laboratory Medicine and Pathobiology, University of Toronto

Toronto, Ontario

**Henry Krieger** **md frcpc**

Specialty: Internal Medicine, Hematology (Medical Oncology)

Staff Member, The Scarborough Hospital—General Division, Sunnybrook and Women’s College Health Sciences Centre

Lecturer, University of Toronto

Toronto, Ontario

**George Kutas** **md frcpc**

Specialty: Internal Medicine (Hematology)

Active Staff Member, Sunnybrook and Women’s

College Health Sciences Centre

Associate Professor, University of Toronto Physician, Toronto–Sunnybrook Regional Cancer Centre

Toronto, Ontario

**Sylvie Lachance** **md frcp**

Specialty: Internal Medicine, Hematology

Staff Hematologist–Oncologist, Stem Cell Transplant Unit, Montreal General Hospital and Royal Victoria Hospital

Associate Professor, McGill University

Montreal, Quebec

**Wendy Lam** **Bs****c (****P****harm)** **md frcpc**

Specialty: Internal Medicine, Hematology

Hematologist/Medical Oncologist, Burnaby Hospital Regional Cancer Centre

Burnaby, British Columbia

**Loree Larratt** **md frcpc**

Specialty: Internal Medicine, Hematology Staff Member, University of Alberta Hospital—Stollery Children’s Centre WCM, Cross Cancer Institute

Associate Professor, University of Alberta

Edmonton, Alberta

**Brian Leber** **md frcpc**

Specialty: Internal Medicine, Hematology

Attending Physician, Hamilton Health Sciences—McMaster University Medical Centre

Associate Professor, Medicine, and Associate Member, Biochemistry, McMaster University

Hamilton, Ontario

**Heather Leitch** **md Phd frcpc**

Specialty: Internal Medicine, Hematology

Hematologist, St. Paul’s Hospital

Clinical Associate Professor, University of British Columbia

Vancouver, British Columbia

**Charles Li** **md frcpc**

Specialty: Internal Medicine, Hematology

Staff Member, St. Paul’s Hospital, University of British Columbia

Clinical Assistant Professor, Department of Medicine, University of British Columbia

Vancouver, British Columbia

**Michel Maheu** **md frcpc**

Specialty: Internal Medicine, Hematology/Pathology, Medical Oncology (QC)

Active Staff Member, Centre Hospitalier Pierre–Le Gardeur

Lachenaie, Quebec

**Kelly McNeil** **Bsc Clsp** **(****mb****)**

Senior Molecular Technologist, Cancer Genetics

Laboratory, B.C. Cancer Agency

Vancouver, British Columbia

**Jacinta Meharchand** **mb Chb frcpc**

Specialty: Internal Medicine, Hematology, Oncology

Division Director, Hematology–Oncology, Toronto

East General Hospital

Toronto, Ontario

**Krishan Mohindra** **md frcpc**

Specialty: Internal Medicine, Hematology

Active Staff Member, Peterborough Regional

Health Centre

Peterborough, Ontario

**Jean–Pierre Moquin** **md frcpc**

Specialty: Internal Medicine, Hematology, Medical Oncology (QC)

Staff Member, Hôpital du Sacre-Coeur de Montréal

Associate Professor, Université de Montréal

Montreal, Quebec

**Thomas Nevill** **md frcpc**

Specialty: Internal Medicine, Hematology (Bone Marrow Transplant)

Active Staff Member, Vancouver Hospital and Health Sciences Centre, British Columbia Cancer Agency—Vancouver Cancer Centre

Associate Professor, University of British Columbia

Vancouver, British Columbia

**Michael Noble** **md frcpc C****ert****ABIM** **(****M****edicine) CertABIM (Hematology)**

Specialty: Internal Medicine, Hematology (Hematology/Oncology)

Active Staff Member, Royal Columbian Hospital, Eagle Ridge Hospital and Health Care Centre

Clinical Assistant Professor, University of British Columbia

Vancouver, British Columbia

**Harold J. Olney** **md frcpc**

Specialty: Internal Medicine, Hematology (QC), Medical Oncology

Staff Member, Centre Hospitalier de l’Université

de Montréal Hôpital Notre-Dame

Montreal, Quebec

**Jaroslav Prchal** **md frcpc**

Specialty: Hematology, Medical Oncology (QC)

Chief, Department of Oncology, St. Mary’s Hospital

Active Staff Member, McGill University Health Centre—Royal Victorial Hospital

Associate Professor, McGill University

Montreal, Quebec

**Yasmin Rahim** **md frcpc facp**

Specialty: Internal Medicine, Hematology, Medical Oncology

Staff Member, Department of Medical Oncology, Toronto East General and Orthopaedic Hospital Inc. Part-time Staff Member, Department of Hematology, Sunnybrook Health Science Centre

Part-time Teaching Staff Member, University of Toronto

Toronto, Ontario

**Marcel Rochon** **md**

Specialty: Hematology (QC), Medical Oncology (QC)

Member, Department of Hematology, Centre Hospitalier Universitaire de Sherbrooke—Hôpital Fleurimont

Consultant, Centre Hospitalier Universitaire de

Sherbrooke—Hôpital Hôtel-Dieu

Sherbrooke, Quebec

**Sheldon Rubin** **md frcpc**

Specialty: Internal Medicine, Hematology

Active Staff Member, Department of Internal Medicine, The Moncton Hospital

Courtesy Staff Member, Dr. Georges-L. Dumont Regional Hospital

Moncton, New Brunswick

**Morel Rubinger** **md frcpc**

Specialty: Internal Medicine, Hematology, Medical Oncology

Staff Member, Department of Medical Oncology, Health Sciences Centre

Associate Professor, Department of Internal Medicine, University of Manitoba

Acting Director, Blood and Marrow Transplant Program—Manitoba

Chair, Lymphoma Working Group

Winnipeg, Manitoba

**Mitchell Sabloff** **md frcpc**

Specialty: Haematology

Staff Member, The Ottawa Hospital—General

Campus Ottawa, Ontario

**Sandeep Sehdev** **md frcpc**

Specialty: Internal Medicine, Medical Oncology (Hematology)

Staff Member, William Osler Health Centre—Brampton Memorial Hospital Campus

Brampton, Ontario

**April Shamy** **md frcpc**

Specialty: Internal Medicine, Hematology

Staff Member, Sir Mortimer B. Davis Jewish General Hospital

Assistant Professor, Department of Medicine, McGill University

Montreal, Quebec

**David Sheridan** **md frcpc**

Specialty: Internal Medicine, Hematology

Professor, Departments of Medicine and Pathology, University of Saskatchewan

Saskatoon, Saskatchewan

**Chaim Shustik** **md**

Specialty: Internal Medicine, Hematology (QC), Medical Oncology (QC)

Attending Physician, McGill University Health Centre—Royal Victoria Hospital

Professor of Medicine, McGill University

Montreal, Quebec

**Raynald Simard** **md frcpc**

Specialty: Internal Medicine, Hematology, Medical Oncology (QC)

Active Staff Member, Department of Hematology, Complexe Hospitalier de la Sagamie

Chicoutimi, Quebec

**Denis Soulières** **md Msc frcpc**

Specialty: Hematology, Medical Oncology

Staff Member, Centre Hospitalier de l’Université

de Montréal—Hôpital Notre-Dame

Director, Hematology Laboratory of Molecular Biology and Special Hematology

Assistant Professor, Université de Montréal

Montreal, Quebec

**Douglas Stewart** **md frcpc**

Specialty: Internal Medicine, Medical Oncology

Staff Member, Tom Baker Cancer Centre, Foothills Medical Centre

Associate Professor, University of Calgary

Calgary, Alberta

**Claude Tessier** **md**

Specialty: Hematology (QC), Medical Oncology (QC) (Ovarian Disorders)

Staff Member, Centre Hospitalier Universitaire de Québec—Hôtel-Dieu de Québec

Active Staff Member, Hôtel-Dieu de Lévis

Quebec City, Quebec

**Cynthia Toze** **md frcpc abim abc** **(Internal Medicine)** **mhs****c (Epidemiology)**

Specialty: Internal Medicine, Hematology

Active Staff Member, Division of Hematology, Vancouver Columbia Cancer Agency, Vancouver Hospital and Health Sciences Centre

Associate Professor, University of British Columbia

Member, Leukemia/BMT Program of British Columbia

Vancouver, British Columbia

**Robert Turner** **md mdcm frcpc**

Specialty: Hematology/Oncology

Staff Member, Clinical Hematology, University of Alberta Hospital

Senior Specialist, Cross Cancer Institute

Professor, University of Alberta

Edmonton, Alberta

**Paul D. Walde** **md frcpc lrcp mrcs mrcp abim**

Specialty: Internal Medicine, Medical Oncology

Active Consulting Staff Member, Algoma District Cancer Program, Sault Area Hospitals

Sault-Ste-Marie, Ontario

**Parveen Wasi** **md frcpc**

Specialty: Internal Medicine, Hematology

Staff Member, Hamilton Health Sciences—McMaster Children’s Hospital

Associate Professor, Department of Medicine, McMaster University

Hamilton, Ontario

**Hilary Wass** **md frcpc**

Specialty: Hematology

Staff Member, British Columbia Cancer Agency—Vancouver Island Cancer Centre

Staff Member, Vancouver Island Health Authority

Victoria, British Columbia

**Jonathan Wilson** **md frcpc facp**

Specialty: Internal Medicine, Hematology, Medical Oncology

Staff Member, Humber River Regional Hospital—Church Site

Courtesy Staff Member, University Health Network—Toronto Western Hospital Site, West Park Healthcare Centre

Lecturer, University of Toronto

Toronto, Ontario

**Anargyros Xenocostas** **md frcpc**

Specialty: Internal Medicine, Hematology

Assistant Professor of Medicine and Consultant in Hematology, London Health Sciences Centre and the University of Western Ontario

London, Ontario

**Sean Young** **Phd**

Laboratory Geneticist, Cancer Genetics Laboratory, B.C. Cancer Agency

Vancouver, British Columbia

## Appendix b CML FACULTY/STEERING COMMITTEE

**Pierre Laneuville** **md frcpc** (Committee Chair)*

Specialty: Internal Medicine, Hematology (Hematology/Oncology)

Division of Hematology, McGill University Health Centre—Royal Victoria Hospital

Associate Professor, Department of Medicine and Oncology, McGill University

President, Canadian Hematology Society Montreal, Quebec

Chair, Quebec and Atlantic Provinces Regional Committee

**Michael Barnett** **bm frcpc frcp Frcpath***

Specialty: Hematology

Head of Hematology, University of British Columbia and Vancouver General Hospital

Member, Leukemia/Bone Marrow Transplant Program of British Columbia, British Columbia Cancer Agency, and Vancouver General Hospital

Vancouver, British Columbia

Chair, Western Provinces Regional Committee

**Robert Bélanger** **md**

Specialty: Hematology (QC), Hematology/Pathology

Staff Member, Department of Hematology, Hôpital Maisonneuve-Rosemont

Assistant Clinical Professor, Université de Montréal Montreal, Quebec

Member, Quebec and Atlantic Provinces Regional Committee

**Stephen Couban** **md frcpc**

Specialty: Internal Medicine, Hematology

Director, Blood and Marrow Transplant Program

Queen Elizabeth II Health Sciences Centre

Associate Professor, Dalhousie University

Halifax, Nova Scotia

Member, Quebec and Atlantic Provinces Regional Committee

**Donna Forrest** **md frcpc**

Specialty: Internal Medicine, Hematology

Associate Member, Leukemia/BMT Program of British Columbia, British Columbia Cancer Research Centre

Staff Member, Vancouver Hospital and Health Sciences Centre

Vancouver, British Columbia

Member, Western Provinces Regional Committee

**Hans Messner** **Phd md frcpc**

Specialty: Internal Medicine

Staff Member, Ontario Cancer Institute, University Health Network—Princess Margaret Hospital Site

Professor, Department of Medicine, University of Toronto

Toronto, Ontario

Member, Ontario Regional Committee

**Denis-Claude Roy** **md frcpc abim abim** **(****HEMATOLOGY****)**

Specialty: Internal Medicine, Hematology (Leukemia and Lymphoma)

Director, Cellular Therapy Laboratory and Program, Hôpital Maisonneuve–Rosemont

Professor of Medicine, Université de Montréal

Montreal, Quebec

Member, Quebec and Atlantic Provinces Regional Committee

**Jeffrey H. Lipton** **Phd md frcpc***

Specialty: Internal Medicine, Medical Oncology (Bone Marrow Transplant)

Head, Chronic Myelogenous Leukemia Group, and Member, Allogeneic Blood and Marrow Transplant Unit, University Health Network—Princess Margaret Hospital Site

Associate Professor of Medicine, University of Toronto

Toronto, Ontario

Chair, Ontario Regional Committee

## Appendix c LEVELS OF EVIDENCE

In keeping with the National Cancer Institute of Canada’s practice of evaluating scientific evidence using the guidelines of recognized organizations, such as the Canadian Task Force on Preventive Health Care and the United States National Cancer Institute, the Canadian Consensus Group on the Management of Chronic Myeloid Leukemia evaluated the evidence according to these classification systems.

### C.1 Research Design Rating

The Research Design Rating set forth by the Canadian Task Force of Preventive Health Care assigns a rating of i to iii as presented in the table that follows ^A^:

**Table:**

*Rating*	*Levels of evidence*
I	Evidence from one or more randomized controlled trials
II-1	Evidence from one or more controlled trials without randomization
II-2	Evidence from cohort or case–control analytic studies, preferably from more than one centre or research group
II-3	Evidence from comparisons between times or places with or without the intervention; dramatic results in uncontrolled experiments could be included here
III	Opinions of respected authorities, based on clinical experience; descriptive studies or reports of expert committees

### C.2 Therapeutic Endpoints

Similarly, the United States National Cancer Institute ranks human cancer treatment studies reporting one or more therapeutic endpoints on a scale in decreasing order of strength from 1iA to 4 according to the following criteria ^B^:

### Strength of study design

1. **Randomized controlled clinical trials** Studies in which participants are assigned by chance to separate groups for the comparison of different treatments. It is the patient’s choice to be in a randomized trial, but neither the researchers not the patient can choose the group in which the patient will be placed. Using chance to assign people helps to ensure that the groups will be similar and that the treatments they receive can be compared objectively. At the time of a trial, there is uncertainty about which of the treatments is best. These trials can be “double-blinded” or “non-blinded.” Double-blinded trials have a stronger study design.
  - *Double-blinded* Neither the patients nor the researchers know which patients are receiving the therapy under study or the comparison (i.e., control) treatment.
  - *Nonblinded* The researchers and the patients know which treatment is being given.
2. **Nonrandomized controlled clinical trials** Studies in which participants are assigned to a treatment group based on criteria that may be known to the researchers, such as the patient’s birth date, chart number, or day of clinic appointment. With this type of study design, there is less confidence that the group receiving the treatment under study and the control group are comparable.
3. **Case series** Studies that describe results from a group or series of patients who all received the treatment that is being investigated. These studies have a weak design, due in part, to the absence of a control group. Types of case series, in descending order of strength, are these:
  - *Population-based, consecutive case series* The study population is well-defined and is either the entire population of interest or a representative random sample of the larger population from which it is drawn. The study subjects receive treatment in the order in which they are identified by the researcher.
  - *Consecutive case series* Studies describing a series of patients who were not limited to a specific population and who received treatment in the same order in which they were identified by the researchers.
  - *Non-consecutive case series* Studies describing a series of patients who were not limited to a specific population and who do not represent a consecutive series of patients identified and treated by the researchers.

### Strength of endpoints measured

- **Total mortality** The proportion of the study population that dies. Frequently called the *death rate.* Measured from a defined point in time, such as the time of diagnosis or the time since treatment was initiated. This is the most easily defined and objective endpoint. The inverse of total mortality, that is, overall survival, may be the reported value.
- **Cause-specific mortality** Death from a specified cause in the population under study, for example, death from cancer versus death from side effects of therapy versus death from other causes. This endpoint is more subjective than total mortality. When death from disease (cancer, heart disease, etc.) is the measured endpoint, the inverse value, that is, disease-specific survival, may be reported instead.
- **Carefully assessed quality of life** Although a very subjective endpoint, quality of life is an extremely important endpoint to patients. The strength of a quality of life assessment depends on the validity of the instruments—that is, the questionnaires, psychological tests, and so on—used.
- **Indirect surrogates** These are measures that substitute for actual health outcomes, and they are subject to investigator interpretation. In descending order of strength, indirect surrogates include these measures:
  - *Disease-free survival* Length of time no cancer was detected after treatment.
  - *Progression-free survival* Length of time disease was stable or did not get worse after treatment.
  - *Tumour response rate* The proportion of patients whose tumours responded to treatment and the degree or extent to which the tumours responded.

### C.3 References

- Canadian Task Force of Preventive Health Care. *Research Design Rating* [online]. London, ON: Canadian Task Force of Preventive Health Care; n.d. [Available at: www.ctfphc.org/ctfphc&methods.htm#Table_2; cited September 2005]
- United States, National Institutes of Health, U.S. National Cancer Institute. *Levels of Evidence for Human Studies of Cancer Complementary and Alternative Medicine (PDQ).* Health Professional Version [online]. Bethesda: U.S. National Cancer Institute; October 2, 2002. [Available at: www.cancer.gov/cancertopics/pdq/levels-evidence-cam/healthprofessional/allpages/print; cited September 2005]
